# Supplementary material for: A UMLS-based spell checker for natural language processing in vaccine safety
Source: BMC Med Inform Decis Mak. 2007 Feb 12;7:3. doi: 10.1186/1472-6947-7-3 (PMC1805499; doi:10.1186/1472-6947-7-3)
Supplement: Additional file 13 — RAP application directory. Sets up directory for RAP (RDF application for PHP) source code [file 1472-6947-7-3-S13.gz › rap/doc/phpdoc/__filesource/fsource_default__vocabularyOWL_RES.php.html]

xml version="1.0" encoding="iso-8859-1"?


File Source for OWL\_RES.php


# Source for file OWL\_RES.php

Documentation is available at OWL\_RES.php

```
1. <?PHP
2. // ----------------------------------------------------------------------------------
3. // OWL Vocabulary (ResResource)
4. // ----------------------------------------------------------------------------------
5. // Version                   : 0.9
6. // Authors                   : Daniel Westphal (dawe@gmx.de)
7. //
8. // Description               : Wrapper, defining resources for all concepts of the Web
9. //                             Ontology Language (OWL). For details about OWL see:
10. //                             http://www.w3.org/TR/owl-ref/
11. //                                Using the wrapper allows you to define all aspects of
12. //                             the language in one spot, simplifing implementation and
13. //                             maintainence.
14. //
15. // ----------------------------------------------------------------------------------
16. // <BR><BR>History:<UL>
17. // <LI>06-25-2004             : $OWL_equivalentClass, $OWL_equivalentProperty, $OWL_Thing, $OWL_Nothing,
18. //                              $OWL_AllDifferent, $OWL_distinctMembers added (auer@informatik.uni-leipzig.de)</LI>
19. // <LI>05-24-2004              : $OWL_DeprecatedClass, $OWL_DeprecatedProperty, $OWL_priorVersion,
20. //                                $OWL_backwardCompatibleWith, $OWL_incompatibleWith (auer@informatik.uni-leipzig.de)</LI>
21. // <LI>03-26-2004              : $OWL:_AnnotationProperty and $OWL_DataRange added (auer@informatik.uni-leipzig.de)</LI>
22. // <LI>02-21-2003             : Initial version</LI>
23. // ----------------------------------------------------------------------------------


29. class OWL_RES{

31. // OWL concepts

33. function ANNOTATION_PROPERTY()
34. {
35. return  new ResResource(OWL_NS . 'AnnotationProperty');

37. }

39. function ALL_DIFFERENT()
40. {
41. return  new ResResource(OWL_NS . 'AllDifferent');

43. }

45. function ALL_VALUES_FROM()
46. {
47. return  new ResResource(OWL_NS . 'allValuesFrom');

49. }

51. function BACKWARD_COMPATIBLE_WITH()
52. {
53. return  new ResResource(OWL_NS . 'backwardCompatibleWith');

55. }

57. function CARDINALITY()
58. {
59. return  new ResResource(OWL_NS . 'cardinality');

61. }

63. function OWL_CLASS()
64. {
65. return  new ResResource(OWL_NS . 'Class');

67. }

69. function COMPLEMENT_OF()
70. {
71. return  new ResResource(OWL_NS . 'complementOf');

73. }

75. function DATATYPE()
76. {
77. return  new ResResource(OWL_NS . 'Datatype');

79. }

81. function DATATYPE_PROPERTY()
82. {
83. return  new ResResource(OWL_NS . 'DatatypeProperty');

85. }

87. function DATA_RANGE()
88. {
89. return  new ResResource(OWL_NS . 'DataRange');

91. }

93. function DATATYPE_RESTRICTION()
94. {
95. return  new ResResource(OWL_NS . 'DatatypeRestriction');

97. }

99. function DEPRECATED_CLASS()
100. {
101. return  new ResResource(OWL_NS . 'DeprecatedClass');

103. }

105. function DEPRECATED_PROPERTY()
106. {
107. return  new ResResource(OWL_NS . 'DeprecatedProperty');

109. }

111. function DISTINCT_MEMBERS()
112. {
113. return  new ResResource(OWL_NS . 'distinctMembers');

115. }

117. function DIFFERENT_FROM()
118. {
119. return  new ResResource(OWL_NS . 'differentFrom');

121. }

123. function DISJOINT_WITH()
124. {
125. return  new ResResource(OWL_NS . 'disjointWith');

127. }

129. function EQUIVALENT_CLASS()
130. {
131. return  new ResResource(OWL_NS . 'equivalentClass');

133. }

135. function EQUIVALENT_PROPERTY()
136. {
137. return  new ResResource(OWL_NS . 'equivalentProperty');

139. }

141. function FUNCTIONAL_PROPERTY()
142. {
143. return  new ResResource(OWL_NS . 'FunctionalProperty');

145. }

147. function HAS_VALUE()
148. {
149. return  new ResResource(OWL_NS . 'hasValue');

151. }

153. function INCOMPATIBLE_WITH()
154. {
155. return  new ResResource(OWL_NS . 'incompatibleWith');

157. }

159. function IMPORTS()
160. {
161. return  new ResResource(OWL_NS . 'imports');

163. }

165. function INTERSECTION_OF()
166. {
167. return  new ResResource(OWL_NS . 'intersectionOf');

169. }

171. function INVERSE_FUNCTIONAL_PROPERTY()
172. {
173. return  new ResResource(OWL_NS . 'InverseFunctionalProperty');

175. }

177. function INVERSE_OF()
178. {
179. return  new ResResource(OWL_NS . 'inverseOf');

181. }

183. function MAX_CARDINALITY()
184. {
185. return  new ResResource(OWL_NS . 'maxCardinality');

187. }

189. function MIN_CARDINALITY()
190. {
191. return  new ResResource(OWL_NS . 'minCardinality');

193. }

195. function NOTHING()
196. {
197. return  new ResResource(OWL_NS . 'Nothing');

199. }

201. function OBJECT_CLASS()
202. {
203. return  new ResResource(OWL_NS . 'ObjectClass');

205. }

207. function OBJECT_PROPERTY()
208. {
209. return  new ResResource(OWL_NS . 'ObjectProperty');

211. }

213. function OBJECT_RESTRICTION()
214. {
215. return  new ResResource(OWL_NS . 'ObjectRestriction');

217. }

219. function ONE_OF()
220. {
221. return  new ResResource(OWL_NS . 'oneOf');

223. }

225. function ON_PROPERTY()
226. {
227. return  new ResResource(OWL_NS . 'onProperty');

229. }

231. function ONTOLOGY()
232. {
233. return  new ResResource(OWL_NS . 'Ontology');

235. }

237. function PRIOR_VERSION()
238. {
239. return  new ResResource(OWL_NS . 'priorVersion');

241. }

243. function PROPERTY()
244. {
245. return  new ResResource(OWL_NS . 'Property');

247. }

249. function RESTRICTION()
250. {
251. return  new ResResource(OWL_NS . 'Restriction');

253. }

255. function SAME_AS()
256. {
257. return  new ResResource(OWL_NS . 'sameAs');

259. }

261. function SAME_CLASS_AS()
262. {
263. return  new ResResource(OWL_NS . 'sameClassAs');

265. }

267. function SAME_INDIVIDUAL_AS()
268. {
269. return  new ResResource(OWL_NS . 'sameIndividualAs');

271. }

273. function SAME_PROPERTY_AS()
274. {
275. return  new ResResource(OWL_NS . 'samePropertyAs');

277. }

279. function SOME_VALUES_FROM()
280. {
281. return  new ResResource(OWL_NS . 'someValuesFrom');

283. }

285. function SYMMETRIC_PROPERTY()
286. {
287. return  new ResResource(OWL_NS . 'SymmetricProperty');

289. }

291. function THING()
292. {
293. return  new ResResource(OWL_NS . 'Thing');

295. }

297. function TRANSITIVE_PROPERTY()
298. {
299. return  new ResResource(OWL_NS . 'TransitiveProperty');

301. }

303. function UNION_OF()
304. {
305. return  new ResResource(OWL_NS . 'unionOf');

307. }

309. function VERSION_INFO()
310. {
311. return  new ResResource(OWL_NS . 'versionInfo');
312. }
313. }


317. ?>
```

Documentation generated on Fri, 17 Dec 2004 16:16:48 +0100 by phpDocumentor 1.3.0RC3
